# Supplementary material for: A pipeline for the reconstruction and evaluation of context-specific human metabolic models at a large-scale
Source: PLoS Comput Biol. 2022 Jun 24;18(6):e1009294. doi: 10.1371/journal.pcbi.1009294 (PMC9278738; doi:10.1371/journal.pcbi.1009294)
Supplement: S1 Appendix — (PDF) [file pcbi.1009294.s003.pdf]

Supplementary Material 2

Predictive performance assessment

The results summarized on Figure 1 depict our large-scale results which show similar predictive accuracy to those reconstructed for MCF7 cell lines. There are slight differences in gene essentiality prediction performance between the 10 selected parameter combinations, with tINIT models reconstructed displaying slightly higher scores. In all of these scenarios, the selected pipeline parameterization choices improved gene essentiality predictions, when comparing with the models reconstructed in the work of Robinson et al, which asserts the importance of using more complex scoring strategies involving global and local thresholds (Figure 1(B)).

The CERES score characterizing gene essentiality in the CRISPR experiments is undoubtedly the parameter that affects these predictions the most, with the best results obtained at a threshold of -0.75. This finding along with the overall low MCC values found with our approaches can be due to several factors. On the one hand, the biomass equation is a generalized assumption of the metabolites needed for cell growth, and thus, is not tailored for each specific cell line. The lack of more exact constraints on model uptake also results in gene knockouts that either do not affect flux through the biomass pseudo-reaction or completely inhibit it, which by itself elicits the usage of a threshold since a direct correlation between CERES scores and growth can not be found using constraint-based models (Figure 1(A)).

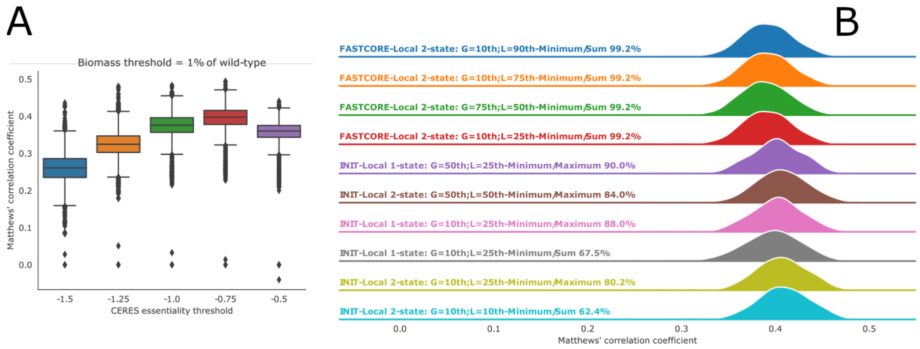

Figure 1: Overview of the predictive capability of the models reconstructed for each CCLE cell line. **A**: MCC value distribution for all models in each lethal gene effect threshold. **B**: Distribution of MCC values for each parameter combination selected for large scale reconstruction of CCLE models. The percentage value at the end of each thresholding strategy description represents the proportion of cell line models that could be successfully reconstructed.
